# Electrospun Polyethylene Terephthalate Nanofibers Loaded with Silver Nanoparticles: Novel Approach in Anti-Infective Therapy

**DOI:** 10.3390/jcm8071039

**Published:** 2019-07-16

**Authors:** Alexandru Mihai Grumezescu, Alexandra Elena Stoica, Mihnea-Ștefan Dima-Bălcescu, Cristina Chircov, Sami Gharbia, Cornel Baltă, Marcel Roșu, Hildegard Herman, Alina Maria Holban, Anton Ficai, Bogdan Stefan Vasile, Ecaterina Andronescu, Mariana Carmen Chifiriuc, Anca Hermenean

**Affiliations:** 1 Department of Science and Engineering of Oxide Materials and Nanomaterials, Faculty of Applied Chemistry and Materials Science, University Politehnica of Bucharest, 060042 Bucharest, Romania; 2 Academy of Romanian Scientists, 050094 Bucharest, Romania; 3 ICUB, Research Institute of Bucharest University, University of Bucharest, 030018 Bucharest, Romania; 4 Faculty of Engineering in Foreign Languages, University Politehnica of Bucharest, 060042 Bucharest, Romania; 5 Institute of Life Sciences, Vasile Goldis Western University of Arad, 310414 Arad, Romania; 6 Microbiology Immunology Department, Faculty of Biology, University of Bucharest, 050107 Bucharest, Romania; 7 Faculty of Medicine, Vasile Goldis Western University of Arad, 310045 Arad, Romania

**Keywords:** polyethylene terephthalate, PET, silver nanoparticles, electrospinning, nanofibers, antimicrobial agents, biocompatibility

## Abstract

Polyethylene terephthalate (PET) is a major pollutant polymer, due to its wide use in food packaging and fiber production industries worldwide. Currently, there is great interest for recycling the huge amount of PET-based materials, derived especially from the food and textile industries. In this study, we applied the electrospinning technique to obtain nanostructured fibrillary membranes based on PET materials. Subsequently, the recycled PET networks were decorated with silver nanoparticles through the chemical reduction method for antimicrobial applications. After the characterization of the materials in terms of crystallinity, chemical bonding, and morphology, the effect against Gram-positive and Gram-negative bacteria, as well as fungal strains, was investigated. Furthermore, in vitro and in vivo biocompatibility tests were performed in order to open up potential biomedical applications, such as wound dressings or implant coatings. Silver-decorated fibers showed lower cytotoxicity and inflammatory effects and increased antibiofilm activity, thus highlighting the potential of these systems for antimicrobial purposes.

## 1. Introduction

Electrospinning is a simple and versatile technique, used to fabricate continuous fibers from a large number of polymers, with diameters ranging from micrometers to several nanometers [1,2,3]. The resulting fibrous mats are characterized by large effective surface areas, continuously interconnected pores, high surface roughness, and usually high porosity [4,5]. It is a highly versatile technique, allowing for the development of structures with various morphologies, including core–shell, hollow, and yarn, only by varying the parameters of the electrospinning, i.e., voltage, feed rate, collector type, distance, and nozzle design [6,7].

Polyethylene terephthalate (PET) is a class of engineered polyesters broadly used in numerous industries owing to its mechanical and thermal properties. PET materials are used in a wide range of applications, such as the automotive industry, filtering membranes, biosensors, protective clothing [8,9,10,11], surgical meshes, drug delivery systems, and tissue engineering scaffolds (i.e., vascular grafts and ligament and tendon substitutes) [11,12,13,14,15,16].

Most of the annual world’s consumption of PET estimated at 13 million tons comes from the packaging industry, raising great concern for environmental pollution [17], with an increasing scientific focus on developing reuse and recycling technologies of PET materials. In this regard, electrospinning is an interesting approach for the fabrication of non-woven nanofiber mats that could reduce environmental waste materials by producing recycled PET materials that could replace previously used materials [18,19]. Moreover, PET electrospun nanofibers could be further implemented in water filtration [20] and heavy-metal adsorption [21] applications. This approach has the potential to significantly decrease the amount of PET waste, by reusing this material in other non-packaging applications.

As reports show that 10% of patients entering an acute hospital develop a healthcare-associated infection, with 9% of cases being surgical wound infections, the risk of wound infections is causing great concern to healthcare professionals [22]. In this context, electrospun nanomaterials are widely investigated for their antimicrobial properties. The common strategy for developing such materials is represented by the attachment or encapsulation of antimicrobial agents, such as antibiotics, cyclodextrins, and metal or metal-oxide nanoparticles onto or into the supporting nanofibers [23]. Silver is the preferred metal oxide used as an antimicrobial agent [24,25,26,27,28,29,30]. Moreover, its nanosized form shows enhanced beneficial properties, owing to the small size, large specific surface area, and quantum effect [31], properties which are correlated with its low toxicity [27,32,33], allowing for the development of diverse applications focused on preventing microbial contamination in different environments or treating microbial infections [34]. There are several studies reporting the uniform incorporation of silver nanoparticles into the electrospun fibers for enhanced antimicrobial abilities [35].

The aim of this study was to obtain electrospun nanostructured fibrillary antimicrobial membranes based on PET materials and silver nanoparticles for different antimicrobial applications. Specifically, the silver nanoparticles were applied onto the surface of the PET materials in order to enrich it with large-spectrum biocidal properties. The different fibers were obtained by adjusting the flow rate of the electrospinning process, since this parameter is very important in ensuring fiber strength and it may impact the morphology and fiber size [36,37]. The in vitro and in vivo biocompatibility issues of PET containing silver nanoparticles were evaluated in order to establish their suitability for biomedical applications. Our goal was to develop a PET-based material that could be further implemented in wound dressing and biomedical coating areas (i.e., implants, medical surfaces, medical textiles), while simultaneously reducing the environmental waste produced by PET usage.

## 2. Experimental Section

### 2.1. Materials

The polyester polymer was obtained from recycled PET bottles available from a local supplier. The bottles previously contained water. Dichloromethane (molecular weight (Mw) = 84.96 g/mol) was purchased from Chimopar trading SRL. Trifluoroacetic acid (Mw = 114.02 g/mol) was obtained from Fluka Analytical. Silver nitrate, NaOH, d-glucose, and eugenol were purchased from Sigma-Aldrich. All chemicals were of analytical purity and used with no further purification.

### 2.2. Electrospinning Deposition of PET Nanofibers

**Scheme 1:**
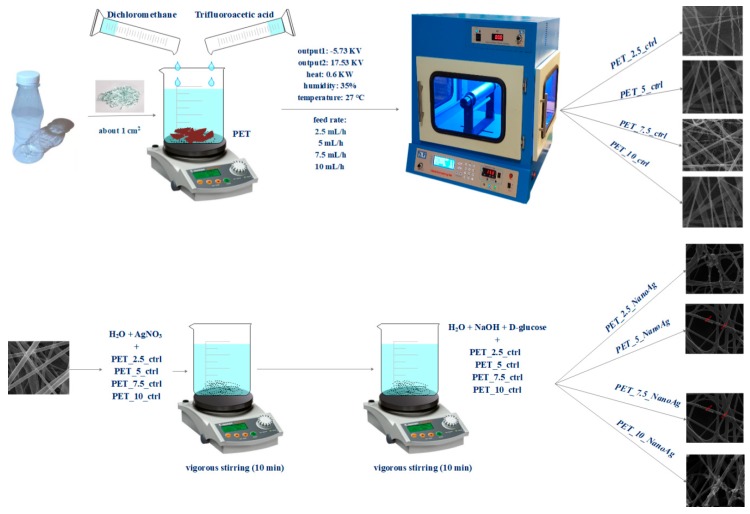
Synthesis process for control and silver-loaded polyethylene terephthalate (PET_X_ctrl and PET_X_NanoAg).

The electrospinning method was used to obtain nanostructured membranes from recycled PET. This method is used to produce membranes made up of a fibrous network with interconnected, overlapping, and randomly distributed fibers. Firstly, the PET bottles were cut into small pieces (about 1 cm^2^) and then added to a mixture of trifluoroacetic acid and dichloromethane. After the complete dissolution of the polymer, electrospinning was performed using the parameters described in Table 1 and Scheme 1.

### 2.3. Silver Nanoparticle (NanoAg) Synthesis

Silver nanoparticles were prepared by reduction. Briefly, two solutions were obtained: one containing 0.1% silver nitrate (0.1 g of AgNO_3_ + 99.9 mL of H_2_O) and one containing the reducing agent (300 mL of H_2_O + 3 g of NaOH + 1 g of d-glucose + 500 uL of eugenol). Sections of the PET’s fibrous networks were placed in the AgNO_3_ solution and left for 10 min under vigorous stirring; subsequently, they were removed and immersed in the reducing agent solution for an equal amount of time. The obtained samples were washed twice with deionized water and allowed to dry at room temperature. After this, the samples were weighed by comparison with the controls in order to estimate the amount of silver nanoparticles immobilized on the fiber surface. The quantified NanoAg varied between 0.998 mg/cm^2^ and 1.003 mg/cm^2^, with an average of measurement ~1 mg/cm^2^ in the case of all samples. Samples coated with silver nanoparticles were noted as PET_2.5_NanoAg, PET_5_NanoAg, PET_7.5_NanoAg, and PET_10_NanoAg.

### 2.4. Physico-Chemical Characterization

**Infrared spectroscopy.** Infrared (IR) spectra were obtained using a Nicolet iN10 MX Fourier-transform (FT)-IR microscope (Thermo Fischer Scientific, Waltham, MA, USA) with a liquid nitrogen-cooled mercury cadmium telluride (MCT) detector with a measurement range of 4000–700 cm^−1^. Spectra collection was performed in reflection mode at a resolution of 4 cm^−1^. For each spectrum, 32 scans were co-added and converted to absorbance using the OmincPicta software (Version 1, Thermo Fischer Scientific, Waltham, MA, USA).

**X-ray diffraction.** Grazing incidence X-ray diffraction (GIXRD) was performed with a Panalytical Empyrean diffractometer (PANalytical, Almelo, The Netherlands), using CuK radiation (1.541874 A) equipped with a 2× Ge (2 2 0) hybrid monochromator for Cu and a parallel plate collimator on the PIXcel3D. Scanning was performed on the 2θ axis in the range of 5–80°, with an incidence angle of 0.5°, a step size of 0.04°, and a time step of 3 s.

**Scanning electron microscopy.** In order to investigate the morphology and size of the nanostructured membranes, images produced by recording the resultant secondary electron beam with an energy of 30 keV of the samples were taken with a scanning electron microscope purchased from FEI (Hillsboro, OR, USA).

**Transmission electron microscopy (TEM). Electron diffraction on selected area.** In order to obtain transmission electron microscopy images, the samples were placed on a carbon-coated copper grid at room temperature. The TEM images were acquired using a high-resolution Tecnai^TM^ G2 F30 S-TWIN transmission microscope equipped with selected area electron diffraction (SAED), purchased from FEI (Hillsboro, OR, USA). This microscope operates in transmission mode at a voltage of 300 kV, while the point and line resolution are guaranteed with values of 2 Å and 1 Å, respectively. The SAED analysis for silver nanoparticles was performed in a light field using the Tecnai^TM^ G2 F30 S-TWIN high-resolution electronic microscope equipped with SAED, purchased from FEI (Hillsboro, OR, USA).

### 2.5. In Vitro Biocompatibility

#### 2.5.1. Cell Line

Human amniotic fluid stem cells (AFSC) were used to evaluate the biocompatibility of the prepared samples. The AFSC cells were cultured in Dulbecco’s modified Eagle’s medium (DMEM) supplemented with 10% fetal bovine serum (FBS) and 1% antibiotics (penicillin, streptomycin/neomycin). All cells were maintained at 37 °C in a humidified incubator with 5% CO_2_.

#### 2.5.2. MTT (3-(4,5-Dimethylthiazolyl-2)-2,5-diphenyltetrazolium bromide) Assay

The evaluation of cell viability was performed by measuring the degree of reduction of a tetrazolium salt solution, MTT (3-(4,5-dimethylthiazolyl-2)-2,5-diphenyltetrazolium bromide), to insoluble purple formazan crystals by viable cells. In this purpose, the AFSC cells were seeded in 96-well plates, with a density of 3000 cells/well under different experimental conditions. Subsequently, a volume of 10 μL of 12 mM MTT was added and the cells were incubated at 37 °C for 4 h. A volume of 100 μL of SDS–HCl solution was further added, vigorously pipetted to solubilize the formazan crystals, incubated for 1 h, and read at 570 nm (TECAN spectrophotometer, Männedorf, Switzerland).

### 2.6. In Vitro Antibacterial Tests

#### 2.6.1. Growth of Planktonic (Floating) Microorganisms in the Presence of Material

To test the effect of the obtained material on the growth of microorganisms in liquid medium (planktonic cultures), the obtained material was cut into 1-cm^2^ samples and sterilized by exposure to ultraviolet (UV) radiation for 30 min on each side. One fragment of the sterile material was individually deposited in a well of a six-well sterile plate. Over the deposited materials, 2 mL of liquid medium and then 20 μL of 0.5 McFarland microbial suspension (bacteria—*Staphylococcus aureus* American Type Culture Collection (ATCC) 25923 and *Pseudomonas aeruginosa* ATCC 27853) or 1McFarland (yeast—*Candida albicans* ATCC 10231) prepared in sterile physiological water, 0.9% NaCl salt, was added to the wells. The as-prepared six-well plates were incubated at 37 °C for 24 h. At the end of the incubation time, 200 μL of the obtained microbial suspension was transferred to 96 sterile plates, and the turbidity of the microbial cultures (absorbance) was spectrophotometrically measured at 600 nm.

#### 2.6.2. Evaluation of Adhesion and Biofilm Formation

To test the effect of fibrillated materials on microbial adhesion and biofilm production, the sterile material samples treated as described above were washed with sterile saline water (SSW), and the medium was changed to allow the microbial cells—adhered onto the surface of the material samples in the first 24 h of incubation—to continue biofilm development and maturation for another 24, 48, and 72 h. After the end of each incubation period, the colonized sample was washed with AFS to remove non-adherent microorganisms and deposited in a sterile tube in 1 mL of SSW. The tube was vigorously vortexed for 30 s and sonicated for 10 s to harvest the cells from the biofilm. The obtained cell suspension was diluted, and various ten-fold serial dilutions were seeded on solid culture media plates in triplicate, to obtain and quantify the number of viable cells, expressed in colony-forming units (CFU)/mL.

### 2.7. In Vivo Biocompatibility Evaluation

#### 2.7.1. Animals and Ethics

CD1 mice were housed in controlled-airflow cabinets with 12-h light cycles and constant temperature and humidity conditions. Animal experiments were performed in accordance with the guidelines of the Vasile Goldis Western University of Arad and approved by the Ethical Committee.

#### 2.7.2. Experimental Design and Surgical Procedures

The mice were randomly assigned to 18 groups (*n* = 5): control, PET_2.5_ctrl, PET_5_ctrl, PET_7.5_ctrl, PET_10_ctrl, PET_2.5_NanoAg, PET_5_NanoAg, PET_7.5_NanoAg, and PET_10_NanoAg, for one day and seven days.

Before the experiment, the material samples (1 cm^2^) were sterilized under UV light for 30 min (both faces) and implanted into a subcutaneous pocket in the dorsum of the animals. For the surgical procedure, the animals were anesthetized by intraperitoneal injection of xylazine/ketamine.

Animals were allowed to recover from anesthesia, housed in individual cages, and observed daily for evidence of wound complications, such as redness, infection, edema, abscess, hematoma, encapsulation, or skin dehiscence.

On days two and seven post-surgery, the animals were euthanatized by anesthetic overdose and the implanted materials, together with the surrounding tissues, were explanted and collected for analysis. Blood was sampled by cardiac puncture to assess acute inflammatory markers, in order to be able to exclude systemic inflammation.

#### 2.7.3. Biochemical Analysis

Venous blood samples were centrifuged at 3500 rpm for 10 min and analyzed for C-reactive protein (CRP) levels, using the CRP FL (ChemaDiagnostica, Monsano, Italy) kit and a Mindray BS-120 Chemistry Analyzer (ShenzenMindray Bio-Medical Electronics Co., Ltd., Nanshan, Shenzhen, China).

#### 2.7.4. Histopathological Analysis

For the histopathological study, explant samples were fixed in phosphate-buffered formaldehyde solution (4%, pH 7.2, 0.05 M), embedded in paraffin, sectioned at 5 μm, and stained with hematoxylin and eosin (H&E) and Gomori’s trichrome kit (Leica Biosystems, 38016SS1, Nussloch, Germany).

Microscopic sections were analyzed with an Olympus BX43 microscope equipped with a digital camera Olympus XC30 and CellSense software and graded for the amount of tissue reaction.

Sections were scored on the degree of inflammatory infiltrate (including acute and chronic inflammatory cells), fibroblasts and neovascularization. Each histometric parameter was graded on a scale of 0–4 for the amount of tissue reaction: − (not present); sp (sporadic) to ++++ (extensive).

#### 2.7.5. Immunohistochemistry

Immunohistochemical studies were performed on paraffin-embedded explant tissue sections of 5 nm thickness, previously deparaffinized and rehydrated using a standard technique. Rabbit polyclonal anti-tumor necrosis factor (TNF)-α diluted 1:100 (Santa Cruz, California) was used as a primary antibody.

Immunoreactions were visualized employing a Novocastra Peroxidase/DAB kit (Leica Biosystems, Nussloch, Germany), according to the manufacturer’s instructions. Negative control sections were processed by the substitution of primary antibodies with irrelevant immunoglobulins of matched isotype used in the same conditions as primary antibodies. Stained slides were analyzed under bright-field microscopy.

### 2.8. Statistical Analysis

Experimental data were statistically evaluated using GraphPad Prism 3.03 software (GraphPad Software, Inc., La Jolla, CA, USA), and one-way analysis of variance, followed by a Bonferroni test. A *p*-value < 0.05 was considered to indicate a statistically significant difference.

## 3. Results

The electrospinning method was used to obtain nanostructured membranes from recycled PET. Different flow rates were used, and the resulted samples were noted according to each flow rate. These samples, prepared at four different flow rates, were further used in combination with silver nanoparticles in order to create alternative biomedical materials. A total of eight different samples were obtained, four with silver nanoparticles and four as controls for in vitro and in vivo tests.

### 3.1. Characterization of the Obtained Materials

**Figure 1:**
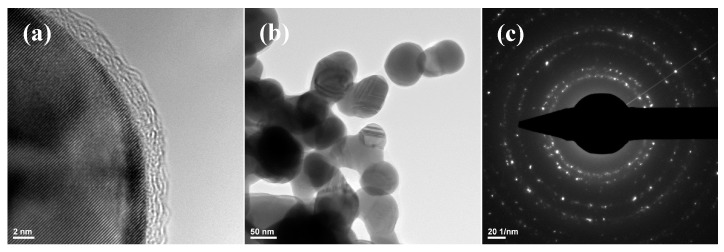
Transmission electron microscopy (TEM) images recorded for (**a**,**b**) silver nanoparticles (NanoAg) and selected area electron diffraction (SAED) pattern (**c**).

Silver nanoparticles obtained through a silver nitrate reduction reaction were characterized by transmission electron microscopy. Figure 1 shows that the size of the obtained particles was in the nanoscale range, varying between 25 and 85 nm. The SAED pattern allowed the identification of the crystalline phases present in the sample, with NanoAg being the only crystalline phase.

**Figure 2:**
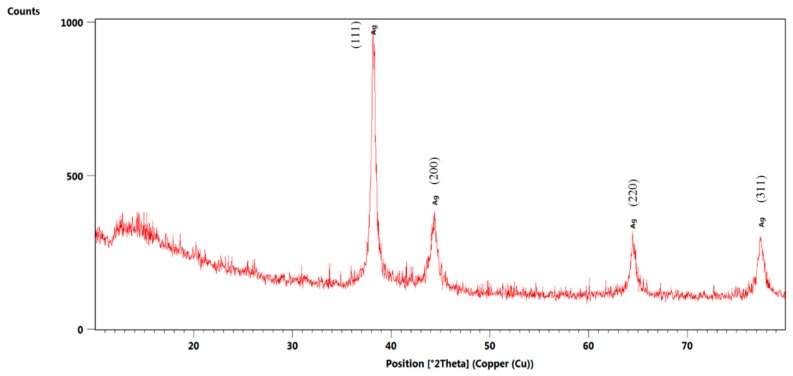
X-ray diffractogram recorded for NanoAg.

Subsequently, NanoAg was characterized by X-ray diffraction. Figure 2 highlights the crystallinity of the synthesized nanoparticles, with the only identified crystalline phase being NanoAg through the four diffraction interferences characteristic of silver nanoparticles.

**Figure 3:**
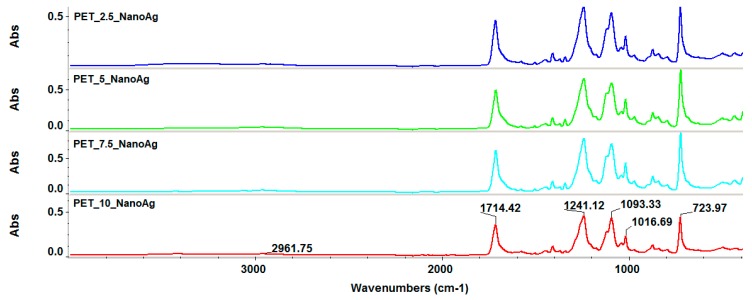
Fourier-transform infrared (FT-IR) spectra recorded for the silver-loaded polyethylene terephthalate (PET_X_NanoAg) samples.

Infrared spectroscopy was used to evaluate the integrity of functional groups during post-processing by electrospinning. Figure 3 reveals that the four experimental variants did not show functional group degradation, absorption band movements, or significant intensity changes. The PET characteristic absorption bands can be identified as follows: 2961 cm^−1^ characteristic of the C–H bond, and 1714 cm^−1^ characteristic of the C=O group. Also, absorption bands of C–C and C–O bonds were observed in the molecular fingerprint area.

**Figure 4:**
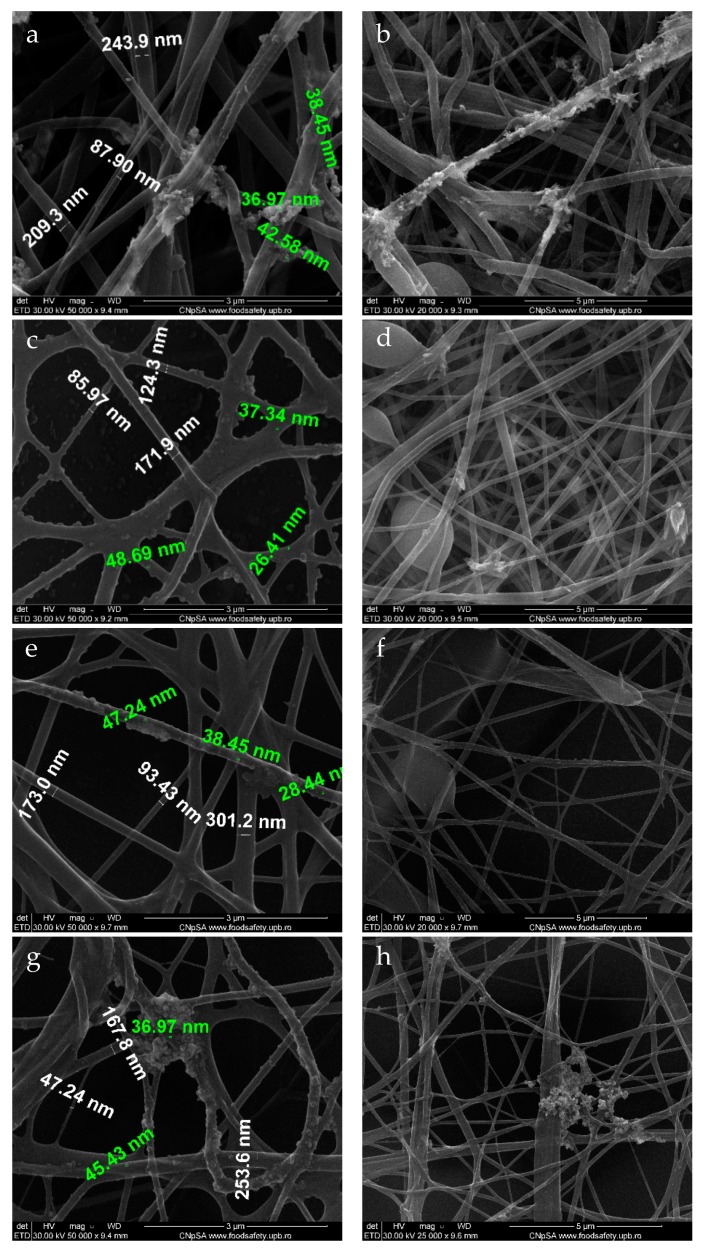
SEM images recorded for PET_X_NanoAg at various flows: (**a**,**b**) PET_2.5_NanoAg; (**c**,**d**) PET_5_NanoAg; (**e**,**f**) PET_7.5_NanoAg; (**g**,**h**) PET_10_NanoAg. Green text—dimensions for nanoAg; white text—dimensions for PET fibers.

Scanning electron microscopy allowed for the identification of the nanostructured membrane morphology and the presence of silver nanoparticles on the fiber surface. Figure 4 reveals the presence of an uneven deposition of silver nanoparticles on the surface of the fibrous membrane in all experimental variants. There was a general tendency of clumping at the nodes of the fibrous network, as the nodes acted as nucleation centers for nanoparticle growth.

**Figure 5:**
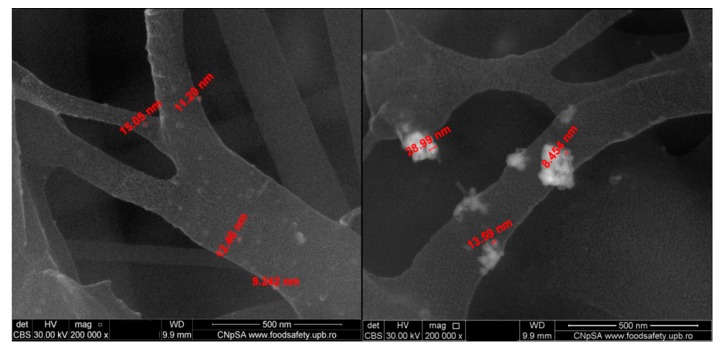
SEM images recorded in backscattering for PET_5_NanoAg.

The electrospun fibers had dimensions ranging between 60 and 250 nm, and the size of the silver particles on the surface of the fibers ranged between 8 and 50 nm. A more detailed representation of the PET_5_NanoAg is presented in Figure 5, highlighting the presence of silver nanoparticles agglomerating at the fiber nodes.

**Figure 6:**
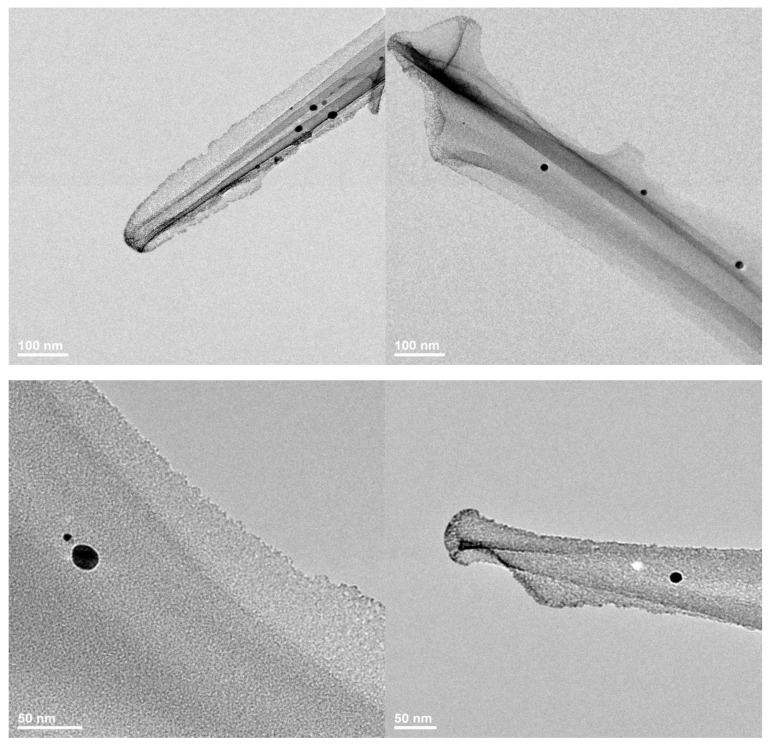
TEM images recorded for PET_2.5_NanoAg.

The nanostructured membranes with modified surface obtained at a flow rate of 2.5 mL/h were also characterized by transmission electron microscopy (Figure 6), which highlighted the nanometric size of the silver particles and their dispersibility over the surface of the PET fibers.

### 3.2. Antimicrobial Properties of the Prepared Samples

Contamination of the environment with undesired microorganisms has negative consequences in different fields, including human health. Microorganisms can grow planktonically, although a great majority of them are adherent to different interfaces and surfaces. Adherent microorganisms are more difficult to remove than microorganisms developing in the planktonic state, due to their ability to form specialized multicellular communities, called biofilms, in which cells may have a different behavior compared to planktonic ones, biochemically and genetically, rendering them more resilient to different stressors. Currently, alternative methods for limiting microbial colonization of raw materials and industrial installations, as well as of biomaterials intended for medical applications, are being studied [38].

In the majority of the experimental variants (except the assays on *C. albicans*), it can be seen that the highest inhibitory activities were obtained in PET samples for which the deposition of fibers by electrospinning was achieved at a flow rate of 10 or 7.5 mL/h (*p*-values ranged from 0–0.05 for *S. aureus* and *P. aerugiosa*).

**Figure 7:**
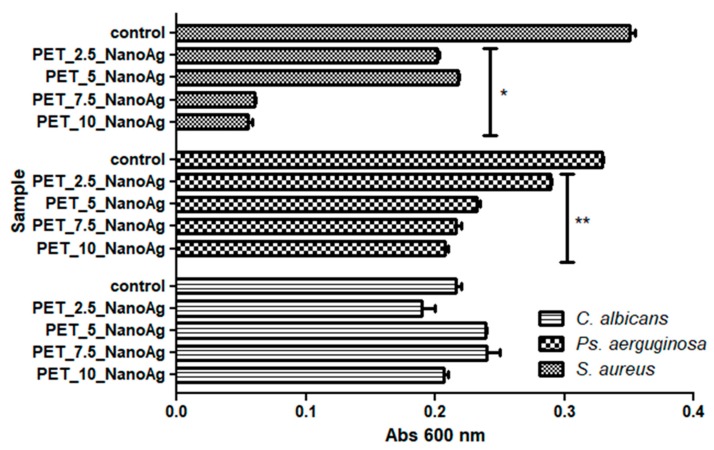
Graphic representation of the recorded absorbance values of *Staphylococcus aureus*, *Pseudomonas aeruginosa*, and *Candida albicans* cultures, expressing the multiplication capacity of these cells after cultivation for 24 h in the presence of recycled PET_X_NanoAg materials. * *p* ≤ 0.001, ** *p* ≤ 0.05 after the comparison of control with NanoAg-containing PET fibers obtained by applying various flow rates).

From the three tested microbial strains, the recycled PET containing NanoAg nanoparticles proved to exhibit the best inhibitory effect on the planktonic growth of *S. aureus* (*p*-value was lower than 0.001), followed by *P. aeruginosa* (*p*-value was lower than 0.05), as compared with the NanoAg free controls (Figure 7).

In the case of the *C. albicans* yeast strain, the inhibitory effect of the planktonic cultures was relatively low, in contrast with the antibacterial one; surprisingly, the most obvious inhibitory effect was observed for PET_2.5_NanoAg, but the result was not statistically significant.

In the case of the assessment of biofilm formation capacity, the results proved to be similar with the data obtained on planktonic cultures, with few variables.

**Figure 8:**
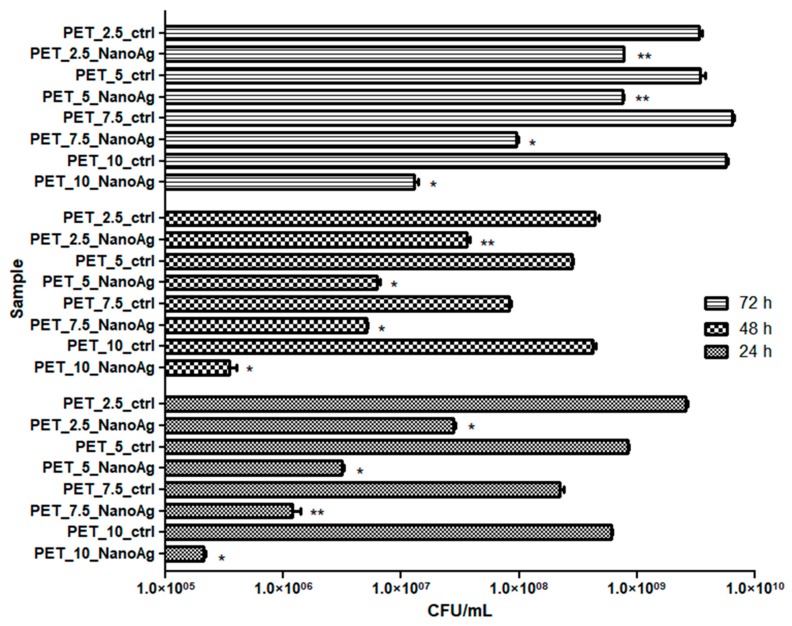
Graphic representation of colony-forming units (CFU)/mL representing the number of *S. aureus* viable cells included in the monospecific biofilms developed on the surface of the materials, quantified after 24 h, 48 h, and 72 h at 37 °C. * *p* ≤ 0.001, ** *p* ≤ 0.05 comparing control PET and NanoAg PET obtained at the same flow rate.

The inhibition effect of *S. aureus* biofilm development was observed at all stages of biofilm development, starting with initial adherence (up to 24 h), continuing with biofilm maturation (up to 48 h) until dispersion (when cells or cell aggregates detach from the biofilm to colonize new surfaces) (Figure 8). The anti-biofilm effect was due to the decrease of viable cells embedded in the biofilm, by 1 to 4 logs, with these data being statistically significant (*p*-values ranged from 0.001–0.05). Similar to the results obtained on planktonic cells, the PET_7.5_NanoAg and PET_10_NanoAg samples also proved to be the most efficient in biofilm inhibition. It can be observed that the anti-biofilm efficiency decreases over the course of biofilm development. This inverse relationship is seen for all four samples, although it is more evident for PET_2.5_NanoAg and PET_5_NanoAg samples (Figure 8).

**Figure 9:**
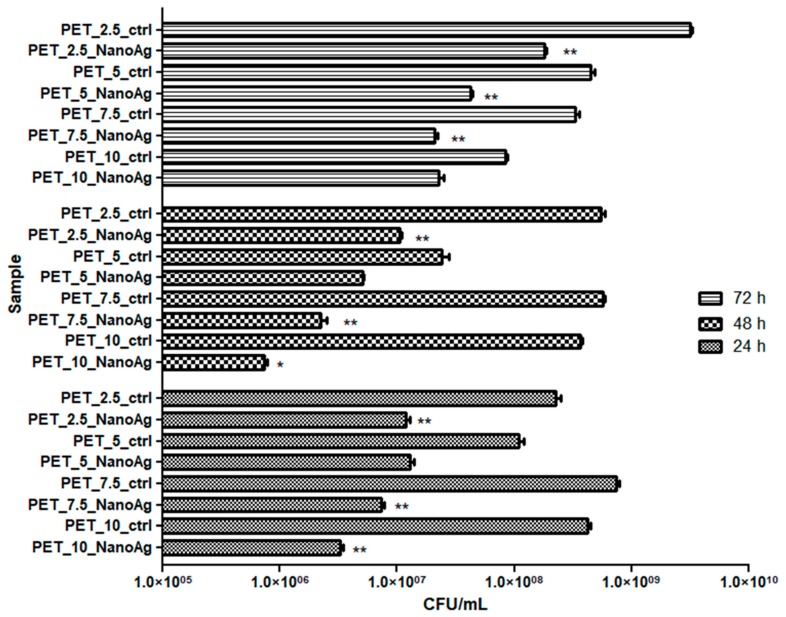
Graphic representation of CFU/mL, representing the number of *P. aeruginosa* viable cells included in the monospecific biofilms developed on the surface of the materials, quantified after 24 h, 48 h, and 72 h at 37 °C. * *p* ≤ 0.001, ** *p* ≤ 0.05 comparing control PET and NanoAg PET obtained at the same flow rate.

*P. aeruginosa* is a microorganism with multiple natural resistance mechanisms, making it an opportunistic pathogen that can colonize with maximum efficiency a great number of environments. Biofilms produced by this opportunistic microorganism are very difficult to eradicate with current antimicrobial substances [39]. The results obtained in this study showed that *P. aeruginosa* has a limited capacity to form biofilms on the obtained nanostructured membranes (Figure 9).

It must be noted that the PET_7.5_NanoAg and PET_10_NanoAg samples proved to be slightly more active against the biofilm formation in *P. aeruginosa*, as compared with the other tested variants. As revealed by Figure 9, the biofilm inhibition capacity of the obtained fibers was maintained relatively constant in all tested time conditions.

**Figure 10:**
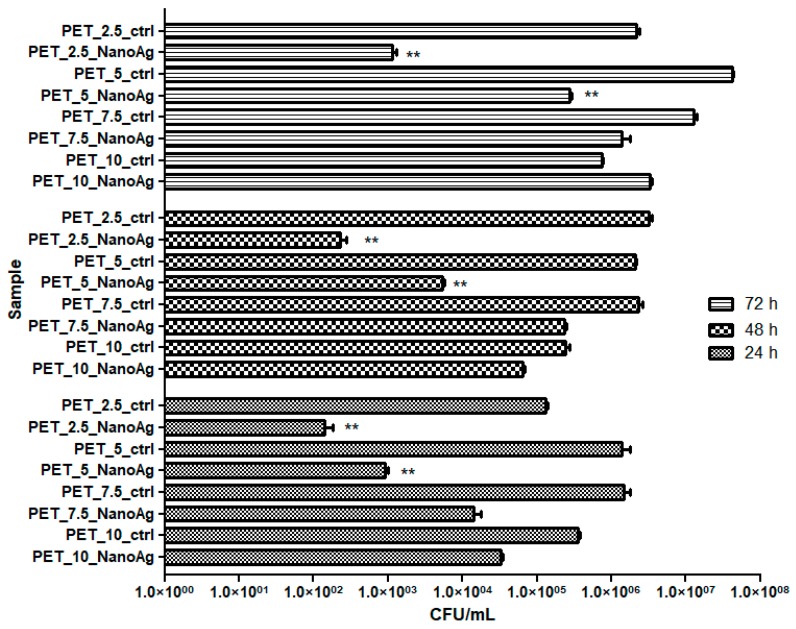
Graphic representation of CFU/mL representing the number of *C. albicans* cells viable cells included in the monospecific biofilms developed on the surface of the materials, quantified after 24 h, 48 h, and 72 h at 37 °C. * *p* ≤ 0.001, ** *p* ≤ 0.05 comparing control PET and NanoAg PET obtained at the same flow rate.

A poor capacity to form biofilms in the presence of the developed nanostructured membranes was observed not only for the bacterial strains, but also for the fungal *C. albicans* strain. In the case of the fungal strain, a dynamic of biofilm growth similar to that observed in case of the Gram-positive *S. aureus* strain was recorded, with an inverse relationship between the age of the biofilm and the intensity of the anti-biofilm effect. However, the efficiency of different tested samples was completely different from that obtained against the two bacterial strains, in the following order: PET_2.5_NanoAg > PET_5_NanoAg > PET_7.5_NanoAg > PET_10_NanoAg (Figure 10).

### 3.3. In Vitro and In Vivo Biological Response

#### 3.3.1. In Vitro Biocompatibility

The cytotoxicity of recycled PET nanostructured membranes was analyzed using human diploid cells in culture. The results obtained by applying the MTT method showed that the proliferation and activity of diploid cells in the culture underwent changes in the presence of the analyzed materials, depending on the rate of deposition of the fibers by electrospinning; additionally, the majority of cases proved that the addition of NanoAg seemed to slightly reduce the cytotoxicity of the obtained materials, as compared with PET controls. However, these results had no statistical relevance (*p*-values were higher than 0.05).

**Figure 11:**
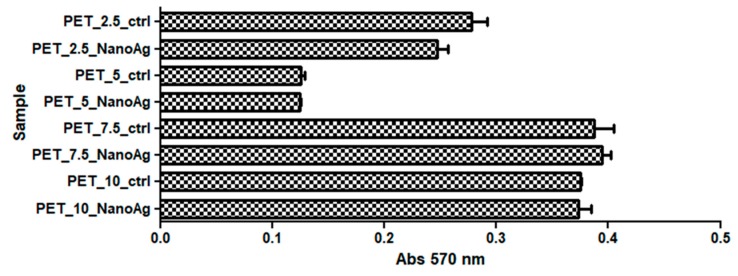
Effects of PET_X_NanoAg on MTT (3-(4,5-dimethylthiazolyl-2)-2,5-diphenyltetrazolium bromide) specific activities in amniotic fluid stem cells (AFSC).

The results obtained by applying the MTT assay revealed that a high percentage of the seeded cells remained metabolically active after covering with NanoAg, suggesting a good biocompatibility of the recycled PET containing NanoAg nanoparticles in vitro (Figure 11).

#### 3.3.2. In Vivo Biocompatibility and Inflammatory Response

**Figure 12:**
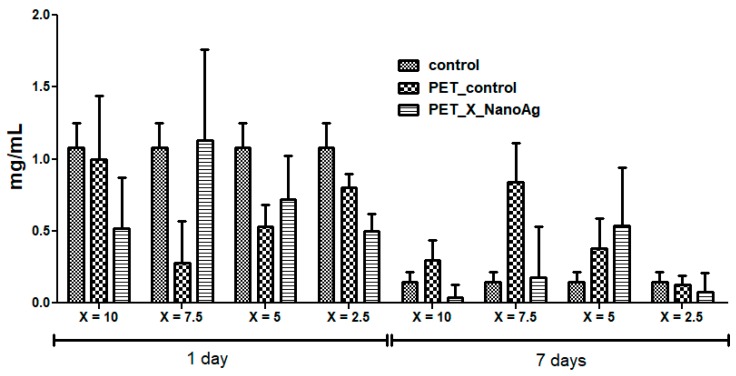
The effects of PET_X_NanoAg subcutaneous implantation in mice on the C-reactive protein (CRP) levels at 24 h and seven days post-surgery.

The subcutaneous implant of the different nanofiber mats showed no adverse local or systemic inflammatory effects. CRP is an inflammation marker, which highlights the activation of a pro-inflammatory cascade. Figure 12 shows the effects of PET_X_NanoAg biomaterials implanted subcutaneously in mice on the CRP serum level. At 24 h post-implantation, the CRP blood level was elevated for all experimental groups, followed by a gradual decrease for up to seven days. Compared to the control (*p* < 0.001), PET_2.5_NanoAg induced significantly lower CRP levels at all time intervals. These results suggest that PET_X_NanoAg biomaterials are well tolerated by the body, and inflammation, together with other potential associated complications, which might lead to implant rejection, is avoided [40,41].

Histopathological analysis of the skin and subcutis of the control animals at days one and seven after implantation showed no significant pathological changes. Inflammatory infiltrate, necrosis, neovascularization, and fibrosis were not observed at either time point. After 24 h post-implantation, PET control samples induced significant edema at the implanting sites, which increased with the rate of fiber deposition. Assessment of inflammatory response revealed the presence of inflammatory cells, such as neutrophils, monocytes, lymphocytes, and macrophages. PET_X_NanoAg samples showed a decreased inflammatory reaction compared with PET-implanted samples at the same rate of electrospinning. In all implants, few eosinophils were noticed (Table 2).

**Figure 13:**
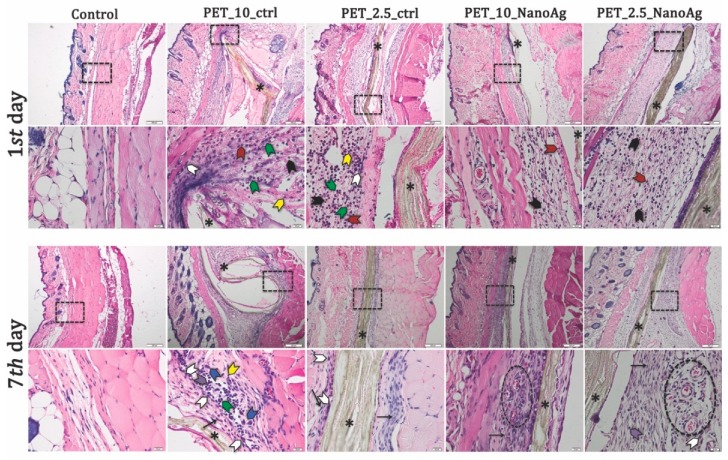
Representative histological images of PET_X_NanoAg mats-implanted sites in mice—days one and seven post-implantation. Neutrophils (black arrowhead); monocytes (green arrowhead); eosinophils (red arrowhead); macrophages (white arrowhead); plasma cells (purple arrowhead); giant cells (blue arrowhead); lymphocytes (yellow arrowhead); * implant (asterisk). Cells were stained with hematoxylin and eosin (H&E) stain. Scale bars = 200 and 20 μm.

On the seventh day post-implantation, the edema reaction persisting in high-purge-speed PET implants and a fibrous capsule of varying thickness were present in all PET-implanted tissue samples (57–76 μm). Consistent with a granulomatous reaction, mainly macrophages, plasma cells, monocytes, lymphocytes, and neutrophils were present at the interface between the mats and this capsule (Figure 13). Some of these macrophages showed marked evidence of phagocytic activity. Giant cells were observed in PET 10 and 7 mL/h samples.

**Figure 14:**
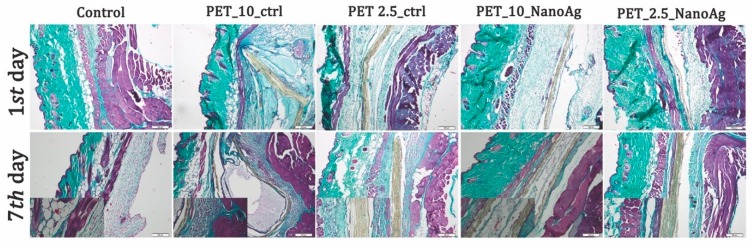
Gomori’s trichrome stain of PET control and PET_X_NanoAg-implanted tissues.

The PET_X_NanoAg samples induced the occurrence of fibrotic capsules (35 and 40 μm) with purge speed with lower thickness as compared with PET in the same electrospinning conditions (Figure 14). Attached cells on the PET_X_NanoAg surface and extensive neovascularization of tissue surrounding the nanofiber mat were noticed.

Injection or implantation of a biomaterial results in an acute inflammation response, which is most often followed by a chronic inflammatory reaction [42], characterized by the infiltration of polymorphonuclear neutrophils (PMN), macrophages, and eventually lymphocytes [43]. The inflammatory reactions toward the novel in situ PET_X_NanoAg materials were weak, being within the limits of a typical, normal reaction to implanted materials characterized by the accumulation of the inflammatory cells on the materials surface. Similar responses were observed in the immediate post-implant period against other implanted materials with increased biocompatibility [44,45].

Implants with prolonged stay in the host tissue generally alter the tissue wound-healing response in chronic inflammatory conditions, producing fibrous encapsulation of the foreign body, with the presence of hallmark giant cells [46]. The fibrous capsule often isolates the implanted materials from normal host tissue sites, being characterized by poor vascularization and reduced bactericidal capability, predisposing these sites to infection [43]. Results from this study showed no well-defined collagen formation around implants in the case of PET_X_NanoAg after seven days post-implantation, a reaction comparable with that induced by other previously reported biocompatible materials [47,48].

**Figure 15:**
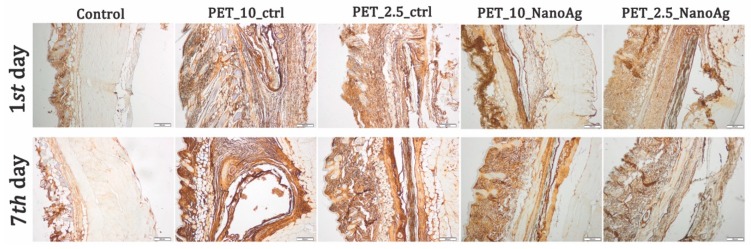
Expression and specific distribution of tumor necrosis factor (TNF)-α at implantation site at 24 h and seven days after implantation; scale bar = 200 μm.

Immunohistochemistry staining was performed for tissue sections to analyze the inflammatory response toward the implanted nanofibers (Figure 15). An increased immunopositivity for TNF-α levels on PET-implanted tissues, as compared to those obtained for the PET_X_NanoAg samples, was observed at both time points.

The progression of events in inflammation and the foreign body response require the extravasation and migration of macrophages to the implant site, which produces and releases platelet-derived growth factor (PDGF), tumor necrosis factor (TNF-α), and interleukin-6 (IL-6) [43]. In our study, the NanoAg covering of PET materials reduced TNF-α expression and consequently reduced inflammation and foreign body response at the implantation site [49,50].

## 4. Discussion

This study focused on the development of antimicrobial fibrous networks consisting of PET materials through the electrospinning technique. With the various applications of electrospun networks, the addition of silver nanoparticles onto the surface of the fibers could potentially be implemented for antimicrobial purposes.

The mechanism of toxicity could be associated with the surface oxidation of the silver nanoparticles and the subsequent release of silver ions, or with the generation of reactive oxygen species and the consequent destabilization of the bacterial membrane [51]. Therefore, an advantage of this study is represented by the formation of the silver nanoparticles on the surface, compared to their direct encapsulation, which could considerably reduce their release. While the material characterization through the GIXRD and FT-IR techniques confirmed the formation of silver nanoparticles on the surface of the PET fibers, a tendency of nanoparticle agglomeration at the nodes was observed in the SEM images. Similar results were previously reported in the literature, either by grafting the nanoparticles on the plasma-treated PET [52] or by using the reduction method [53], and it was stated these clusters could be responsible for a slightly reduced antibacterial property [35,54]. In this regard, there are several solutions reported for enhancing the homogeneous distribution of the silver nanoparticles onto the surface of the fibers, such as sonochemical coating [55], plasma treatment of the silver nitrate-containing polymer solution [56], or chemically reducing the silver nitrate in the polymer matrix prior to the fiber fabrication. Moreover, it was previously reported in the literature that concentrations of silver nanoparticles as low as 0.05% can considerably reduce the incidence of surgery-related infections [57], with demonstrated bacteriostatic effects [58]. Studies reported that the effects of silver nanoparticles are strongly related to the size of the nanoparticles, with 10 nm being related to both the formation of silver ions during dissolution and the penetration of nanoparticles through the bacterial wall [59].

Furthermore, green synthesis methods could also be applied for the production of silver nanoparticles, thus avoiding the use of potentially toxic reducing agents. Examples of such agents can be found in the literature, most commonly involving chitosan [60] and other polysaccharides, plant extracts [61,62,63], or microbial extracts [64], all exhibiting enhanced antibacterial properties. In the present study, the investigated biomaterials proved to be efficient against the three types of bacteria, both in the planktonic and attached states, thus confirming their potential in anti-infective applications.

Despite their wide use as antimicrobial agents, silver nanoparticles are often associated with cytotoxic effects on the normal cells and have a tendency to accumulate in vital organs, such as the spleen, liver, kidney, and brain. Therefore, they must be considered “double-edged swords” for biomedical applications [65]. In this respect, silver nanoparticles are usually embedded in a composite matrix that has a significant role in controlling the release of the silver ions. In this study, the formation of silver nanoparticles on the surface of the PET biomaterials proved to reduce the cytotoxic effects of the uncoated materials determined by the MTT assay. Similarly, the in vivo biocompatibility tests demonstrated the anti-inflammatory potential of the NanoAg samples as compared to the PET samples, as fewer inflammatory cells were present at the implantation site in the former case. It could be hypothesized that the composite material could delay the release of the silver ions and, subsequently, the release of the pro-inflammatory factors, thus improving the potential for the long-term use of these biomaterials. This is in accordance with previous studies, which demonstrated foreign body reactions of PET materials, including commercial sutures for orthopedic purposes [66]. Additionally, there are studies suggesting the influence of the fiber size, mesh porosity, or contact surface, with one group reporting a minimum foreign body reaction of a PET mesh compared to the highly biocompatible bulk PET material [67].

This study could, therefore, represent a starting point for the development of antimicrobial membranes, which could be used as wound dressings or biomedical coatings, as they exhibit good antimicrobial properties and no inflammatory or cytotoxic effects.

## 5. Conclusions

The obtained membranes, composed of recycled PET nanofibers obtained by electrospinning at four different flow rates, decorated with silver nanoparticles, were characterized by GIXRD, FT-IR, SEM, TEM, and SAED. Results proved the formation of the silver nanoparticles on the surface of the fibers, with a slight tendency of agglomeration at the nodes. The dimensions of the fibers varied between 30 and 100 nm, while the silver nanoparticles ranged in size between 8 and 20 nm. Furthermore, the antimicrobial properties of the materials showed good antimicrobial and antibiofilm activity against Gram-positive and Gram-negative bacteria, as well as fungal strains. The biocompatibility tests were performed both in vitro, on human AFSC cells, and in vivo, through the subcutaneous implantation of the fibrous networks in CD1 mice. Results showed acceptable levels of toxicity in the in vitro and in vivo assays, with lower cytotoxic and inflammatory effects for the silver nanoparticle-decorated fibers. These results recommend the obtained materials for different antimicrobial applications, both in industry and in the biomedical field, opening promising new perspectives for the PET materials.

## Figures and Tables

**Table 1:** The parameters utilized for electrospinning. PET—polyethylene terephthalate.

Sample	Output 1 (kV)	Output 2 (kV)	Heat (KW)	Humidity (%)	Temperature (°C)	Feed Rate (mL/h)
PET_2.5_ctrl	−5.73	17.53	0.6	35	27	2.5
PET_5_ctrl						5
PET_7.5_ctrl						7.5
PET_10_ctrl						10

**Table 2:** Tissue reactions by histometric scoring used to grade inflammation, fibrosis, necrosis, and neovascularization in the tissue surrounding subcutaneous implants.

Nanofibers	Explan Tation (days)	Edema	PMN	MONO LYM	MO	GC	PC	EOS	FC	NV
Control mice	1	−	+	−	−	−	−	−	−	−
	7	−	−	+	+	−	−	−	−	−
PET_10_ctrl	1	++++	++++	+++	++	+	−	+	+	−
	7	+++	++	++++	++++	++	++	+	++++	−
PET_7.5_ctrl	1	+++	+++	+	+	+	+	+	+	−
	7	++	+	++	+++	++	+	+	+++	−
PET_5_ctrl	1	+	+++	sp	+	sp	−	+	+	−
	7	++	+	++	+++	+	+	+	++	−
PET_2.5_ctrl	1	+	+++	sp	+	sp	−	+	+	−
	7	++	+	++	+++	+	+	+	++	+
PET_10_NanoAg	1	−	+++	++	++	−	−	+	+	−
	7	−	+	+++	+++	−	+	+	+++	+
PET_7.5_NanoAg	1	−	++	sp	sp	−	+	+	+	−
	7	−	+	++	++	−	+	+	+++	+
PET_5_NanoAg	1	−	++	sp	+	−	−	+	+	−
	7	−	+	+	+++	−	+	+	++	+
PET_2.5_NanoAg	1	−	++	sp	+	−	−	+	+	−
	7	−	+	+	+	−	+	sp	+	++

PMN: polymorphonuclear neutrophils; MONO: monocytes; LYM: lymphocytes; EOS: eosinophils; MO: macrophages; PC: plasma cell; GC: giant cell; FC: fibrocytes, NV: neovascularization. Tissue reactions are rated from − (not present), and sp (sporadic) to ++++ (extensive).
